# The Promotional Effect of Health Education on the Medical Service Utilization of Migrants: Evidence From China

**DOI:** 10.3389/fpubh.2021.818930

**Published:** 2022-01-28

**Authors:** Yihao Tian, Tao Luo, Yuxiao Chen

**Affiliations:** ^1^Department of Public Service Management and Public Policy, School of Public Administration, Sichuan University, Chengdu, China; ^2^Social Development and Social Risk Control Research Center of Sichuan Philosophy and Social Sciences Key Research Base, Chengdu, China; ^3^Department of Public Administration, School of Politics and Public Administration, Zhengzhou University, Zhengzhou, China

**Keywords:** the migrants, health education, medical service utilization, medical service resources, age heterogeneity, education heterogeneity

## Abstract

There were 376 million migrants in China by 2020, who made significant contributions to urban development. However, they used limited medical services and had lower self-reported health status than inflow city residents. Based on this, this study uses the cross-sectional data of the 2017 China Migrants Dynamic Survey (CMDS) to construct a multiple linear regression model to empirically study the role of health education in improving medical services utilization for migrants. It finds that compared to migrants without health education, the probability of the medical service utilization for migrants with health education has increased significantly, and counseling is more effective than other methods for health education. This promotion effect of health education has been established after a series of robustness tests. Furthermore, this study finds that the closer the migrants are to medical service resources, the greater the effect of health education on medical services utilization for migrants. The heterogeneity test shows that the effect of health education on medical services utilization for migrants is greater among the non-elderly and those with lower education levels. From the perspective of health education, the findings in this study provide empirical evidence to support the government in formulating policies to improve the utilization of medical services for migrants and reduce health inequality.

## Introduction

**Figure 1:**
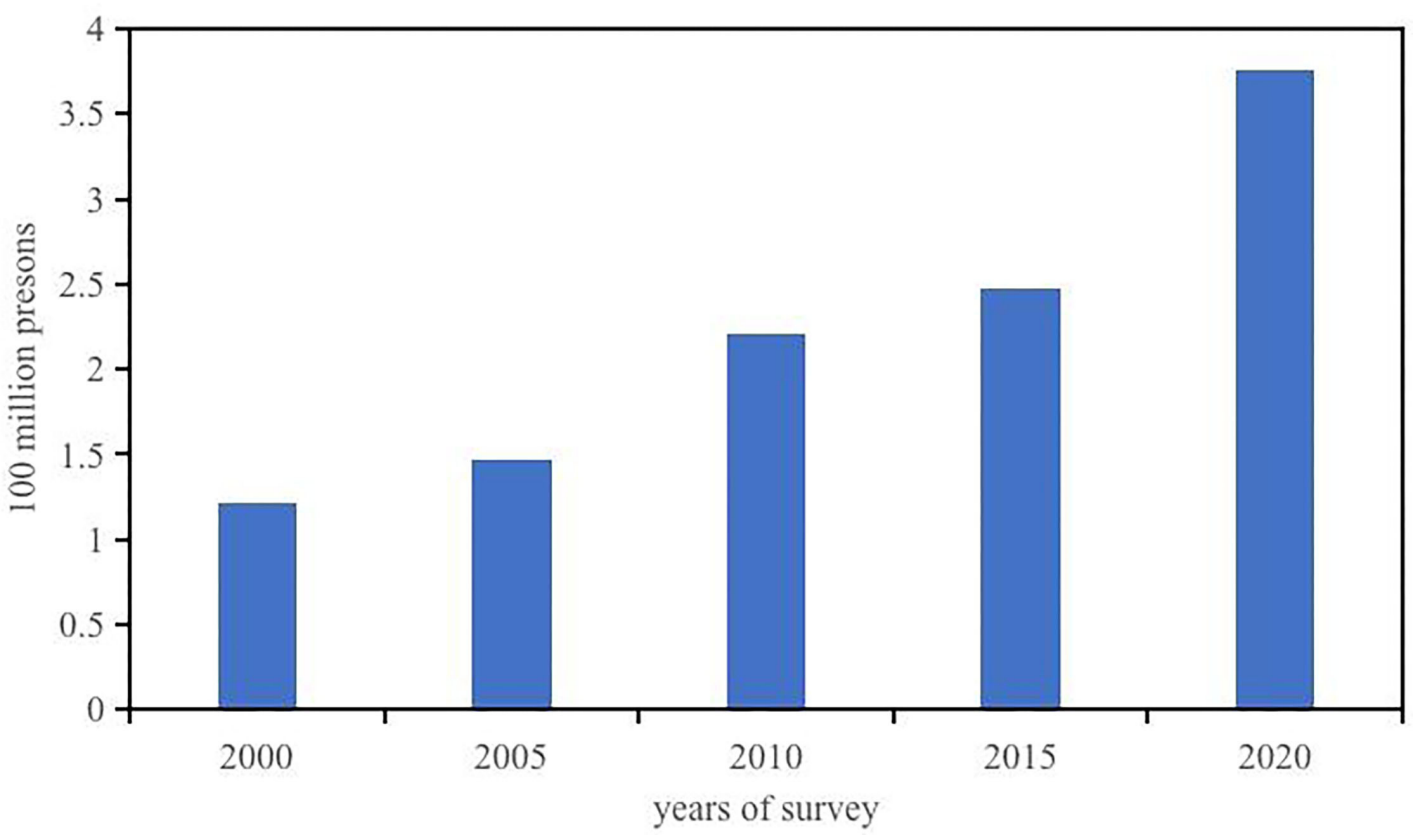
The changing trend of migrants in China. Data sources from National Bureau of Statistics of China.

Migration is an indispensable part of the contemporary social, political, and economic worlds (1). The unprecedented economic growth and urbanization over the past few decades in China has caused a large-scale increase in the number of internal migrants (2–4). As shown in Figure 1, their numbers have reached nearly 376 million, according to China's seventh national census in 2020, and accounting for 27% of China's total population. This means that, on average, one in four households is inhabited by a migrant. However, migrants are discriminated against in many areas of life, such as education, employment, income, social integration (5–12), and particularly health services (13–16). Compared with residents with household registration (Hukou) of the inflow cities, the utilization of medical services by migrants is significantly lower. According to the 5^th^ China National Health Services Survey, the hospitalization rate of the migrants due to illness (excluding childbirth) was 4.0%, much lower than the 8.2% of urban residents and 8.0% of rural residents (17, 18). These migrants have a lower level of education; 83% of them work in construction, catering, and other industries for heavy, high-intensity, long-term work, which increases the risk of illness (19, 20). The risk of diseases and the lower utilization rate of medical services result in the deterioration of the health of migrants and severe health inequality. It is of great significance to explore the utilization of medical services for migrants from different angles, to improve the health of migrants and reduce health inequality.

The three causes of this low utilization of medical services have been summarized in the literature. First, there is a lack of medical insurance in inflow cities. According to the 2017 China Migrants Dynamic Survey (CMDS), only 21.91% of the migrants have medical insurance in the place of inflow. The social insurance system for migrant workers in China has poor portability and fragmentation (21). The medical insurance system in China is coordinated by counties or cities, which means different counties and cities have different medical insurance policies, such as Insurance financing and benefits. If medical services are used outside of where medical insurance is purchased, the reimbursement of medical expenses will be low or even non-reimbursable, and the process will become cumbersome (22). Moreover, owing to the restrictions of the household registration system (Hukou), migrant workers suffer from institutional discrimination in the job market. They have difficulty participating in local social insurance in the inflow cities (22). The social insurance for employees is paid by the firm and the employee in China, with the firm contributing about 80% of the premium. The social insurance cost of employees already accounts for more than 40% of the labor cost of Chinese firms, which is significantly higher than that in other Asian countries (23). Most firms that employ migrants are labor-intensive, and have relatively low profit margins. Social insurance costs increase the operating burden on firms. Consequently, many of these firms choose not to provide insurance to their employees (24–30). Therefore, the lack of medical insurance in inflow cities leads to migrants paying a higher cost to have medical services than local residents, and thus, reducing their utilization.

Second, the demand characteristics of migrants are an important reason for their low utilization of medical services. Migrant workers, who are relatively less educated, mostly work in labor-intensive industries, such as construction and manufacturing, and earn a low income. Due to these factors, they often choose to increase their current income, and do not avail medical services when they get sick (31).

Third, health literacy among migrants is low. With a lack of knowledge about health, migrants prefer to self-heal rather than access medical services when they get sick. They tend to seek medical assistance when their condition worsens (32). This indicates that migrants have low health literacy, hardly pay attention to illnesses, and would not seek medical services even if they could access them.

Health education is considered an important way to improve health literacy (33). Health education refers to a planned, organized, and systematic social education activity to make people consciously adopt behaviors and lifestyles that are beneficial to health, eliminate or reduce factors risking their health, prevent diseases, improve health conditions for better life quality. The Chinese government has proposed the *Action Plan for Health Education and Promotion of Migrants* (2016–2020) to improve health literacy among migrants. The action intends to provide health education to migrants through health departments, communities, enterprises, and schools, including public health, disease prevention, reproductive health, and mental health education. The purpose of this action plan is to increase knowledge about health, enhance health awareness among migrants to improve their health literacy and utilization of medical services, further improve the health of migrants, and, at the same time, promote the implementation of health policies and the construction of a healthy environment. However, can health education be effective in increasing medical service utilization among migrants? How can health education be conducted more effectively? Does the impact of health education vary depending on the accessibility of medical services and certain characteristics of migrants? The current literature does not adequately address these questions.

Based on the above knowledge gap, using data from the CMDS, this study empirically studied the impact of health education on the medical service utilization of migrants with a multiple linear regression model and a logit model, and identified the moderating effect of the accessibility of medical services. It also verified the heterogeneous impact of health education on different age and education groups. This study enriches the understanding of the factors affecting the medical service utilization of the migrants. It is useful for formulating policies that improve the health literacy of migrants and improve migrant's health.

## Methodology

### Data Source

The dataset used in this study comes from the 2017 (CMDS), conducted by the National Health Commission, P.R. China. The CMDS subjects are migrant workers from 31 provinces, autonomous regions, municipalities, and the Xinjiang Production and Construction Corps. These subjects lived in inflow cities for more than 1 month and did not have local Hukou. The data has strong representativeness and a rich set of variables. The survey encompasses the basic characteristics of migrants, and their employment, family members, insurance participation, and health status. After excluding observations with missing key variables, a total of 77,837 observations were obtained.

### Variables

#### Dependent Variable

The dependent variable in this study was the medical service utilization of migrants. In the CMDS, respondents were asked to answer the question, “In the past year, did you use medical services when you had a diarrhea/fever/rash/jaundice/conjunctival swelling/cold?” We established six medical service utilization variables for six different diseases. The answer to “yes” was coded as “1,” defining the utilization of medical service, “no” was coded as “0,” defining the non-utilization of medical service, and “unclear” was coded as a missing value. We conducted a benchmark regression on six medical service utilization variables separately to analyze the effect of health education on the medical service utilization of migrants for different diseases. To study the overall medical service utilization of the migrants, if the respondent had any of the above-mentioned diseases and used medical services, it will be coded as “1,” demonstrating that medical service utilization occurred. Otherwise, it will be coded as “0,” and the respondent without disease will be coded as a missing value.

#### Explanatory Variable

The core explanatory variable in this study was health education. Respondents were asked to answer separately “In the past year, did you receive health education on occupational diseases/AIDS/ reproductive health and contraception/tuberculosis/smoking/mental health/chronic diseases/maternal and child health/self-help in public emergencies?” If the respondent had participated in any health education, it will be coded as “1,” defining that received health education; otherwise it will be coded as “0.”

#### Control Variables

To obtain the effects of health education on the medical service utilization of migrants, we controlled other factors that may affect it, including personal and family characteristics. Personal characteristics include gender, age, education, political identity, and industry type; family characteristics include Hukou, marital status, family size, and household income. And we also controlled medical insurance and native perception. In addition, we controlled the inflow county fixed effects.

##### Gender

The difference in health between men and women has been shown in multiple studies (34–36). Therefore, we controlled the dummy variable for gender in this study (female = 0, male = 1).

##### Age

We calculated the specific age of the respondents as of May 2017, which was the time of the survey, based on their month and year of birth, which is a continuous variable.

##### Education

We converted respondents' education level into years, specifically, less than elementary = 0, elementary = 6, middle school = 9, senior middle school/technical secondary school = 12, undergraduate/junior college = 15, and postgraduate = 19. This is a continuous variable.

##### CPC Identity

In China, the Communist Party of China (CPC) identity is a unique social capital with the collective value of all social networks (37). Migrants with CPC membership have more social capital and fewer barriers to accessing medical services. Therefore, we controlled for the dummy variable for CPC membership (CPC member = 1, others = 0).

##### Industry

We include a dummy variable for the industry in which a migrant works in the model (primary industry = 1; secondary industry = 2; tertiary industry = 3).

##### Hukou

The household registration system (Hukou) has caused a split in the provision of public services, including medical services, between urban and rural areas in China. It is one of the most important reasons for emerging migrants (38–40). We controlled for the dummy variable for the type of Hukou (rural Hukou = 0, others = 1).

##### Marital Status

We divided the marital status of the migrants into married and unmarried. Singles, divorced, widowed, and cohabiting were considered unmarried, and first marriage or remarriage were considered married. This was measured as a dummy variable (unmarried = 0, married = 1).

##### Family Size

It refers to the number of migrant family members. Family members pay attention to and support each other. There is a learning and peer effect within the family, which makes it easier to contact each other, share health-related knowledge, and improve health literacy (41). This is a continuous variable.

##### Household Income

Income is an important determinant of health and medical service utilization (42). China has a strong family concept, and when an illness is encountered, the family shares the burden. Therefore, we controlled household income rather than individual income, which is a continuous variable.

##### Medical Insurance

As mentioned above, medical insurance is a key determinant for the medical service utilization of migrants (43–46). The CMDS surveyed the participation of the migrants in five categories of medical insurance. If the respondent participated in at least one kind of medical insurance, we coded it as “1,” indicating participation in medical insurance; otherwise it will be coded as “0.” This was a dummy variable.

##### Native Perception

The impact of social integration on health has been increasingly emphasized (47–49). Social integration facilitates the utilization of medical services. The question “Do you agree with the statement ‘I feel I am already a native'?” reflects the respondent's subjective level of social integration. The answer of “strongly disagree” was coded as “0,” “disagree” was coded as “1,” “agree” was coded as “2,” and “strongly agree” was coded as “3.”

##### Regional Effects

We controlled for the regional effect at the county level, and the region was a dummy variable measured by the county where respondents inflowed. Compared to previous studies controlling at the provincial level, this increases the reliability of this study.

### Empirical Model

The empirical model in this study is used to test the impact of health education on the medical service utilization of migrants, that is, whether health education for migrants will promote their medical service utilization. The basic model is as follows:


(1) $$\begin{array}{l} {MSU}_{i}=\alpha +\beta {HealthEdu}_{i}+\gamma {X^{\prime }}_{i}+\lambda_{j}+\varepsilon_{i} \end{array}$$

where *MSU*_*i*_ is the dependent variable representing the medical service utilization of migrant *i*. α is a constant. *HealthEdu*_*i*_ is the explanatory variable that this study interested in, representing the health education received by migrant *i*. ${X^{\prime }}_{i}$ represents a series of control variables that may affect the medical service utilization of migrant *i*, including personal characteristics, family characteristics, and other factors. λ_*j*_ indicates the fixed effect of the inflow county, which controls the factors change with county and does not change over time. ε_*i*_ is the error term. We use a two-stage least squares regression with an instrumental variable (IV) to address the problem of endogeneity, and the details are presented in Section results.

In the robustness test, as our dependent variable *MSU*_*i*_ is a dummy variable, we performed a logit model for the robustness test, and the model is as follows:


(2) $$\begin{array}{l} ln\left(\frac{p_{i}}{1-p_{i}}\right)=\alpha +\beta {HealthEdu}_{i}+\gamma {X^{\prime }}_{i}+\lambda_{j}+\varepsilon_{i} \end{array}$$

where, *p*_*i*_ is the probability that medical service utilization occurs after the illness of migrant *i* and 1−*p*_*i*_ is the probability that medical service utilization does not occur. The other variables are the same as in Equation (1).

## Results

### Descriptive Statistics

Descriptive statistics for all variables are shown in Table 1. Regarding the dependent variable, 40.24% of migrants made use of medical services after an illness. Regarding the explanatory variable, 73.09% of migrants received health education. As shown in Table 2, we divided the sample into two subsamples based on the explanatory variable (health education). We performed descriptive statistics separately for the dependent variable (medical service utilization). It was found that, compared to the migrants who did not receive any health education, the mean medical service utilization of the migrants who received health education is higher. It is statistically significant at the 1% level.

**Table 1:** Descriptive statistics.

**Variable**	**Item**	**Freq**.	**Percent**	
Medical service utilization	Yes = 1	41,815	40.24	
	Not = 0	62,089	59.76	
Health education	Yes = 1	112,989	73.09	
	Not = 0	41,597	26.91	
Gender	Male = 1	87,871	51.69	
	Female = 0	82,118	48.31	
CPC identity	Yes = 1	8,463	4.98	
	Not = 0	161,526	95.02	
Industry	Primary industry = 1	3,307	2.36	
	Secondary industry = 2	50,570	36.16	
	Tertiary industry = 3	85,965	61.47	
Marital status	Married = 1	138,083	81.23	
	Unmarried = 0	31,906	18.77	
Hukou	Rural = 0	132,555	77.98	
	Others = 1	37,434	22.02	
Medical insurance	Have = 1	156,071	91.81	
	Not = 0	13,918	8.19	
Native perception	Strongly disagree = 0	5,304	3.12	
	Disagree = 1	35,405	20.83	
	Agree = 2	86,635	50.97	
	Strongly agree = 3	42,645	25.09	
**Variable**	**Mean**	**SD**	**Min**	**Max**
Age (year)	36.99	11.08	16	97
Education (year)	10.11	3.418	0	19
Family size	3.140	1.200	1	10
Household income (per month)	7.136	5.759	−90	200

*The unit of household income is thousand CNY*.

**Table 2:** The gap in medical service utilization between the migrants with and without health education.

**Variable**	**Health education**				***T*-test**
	**Yes**		**No**		
**Medical service utilization**	**Mean**	**Sd**	**Mean**	**Sd**	**Diff**
	0.419	0.493	0.345	0.475	0.074^***^

***, ^**^, and ^*^ represent significance at the 1, 5, and 10% levels, respectively.

Concerning the control variables, 51.69% of the sampled migrants were male. The age of the migrants in the total sample ranges from 16 to 97 years, with a mean age of about 37 years. The average education level of the sampled migrants is 10.11 years, with a minimum of 0 years (illiteracy) and a maximum of 19 years (postgraduate). The percentage of CPC membership among migrants is only 4.98, and 97.64% of migrants work in the secondary and tertiary industries. In terms of marital status, 81.23% of the migrants were married, and 18.77% were unmarried. On average, there are 3.14 members in each migrant family in the sample, with family sizes ranging from 1 to 10. The average monthly household income was 7,136 CNY in 2017. The percentage of rural Hukou among migrants is even larger, reaching 77.98%. We found that most Chinese migrants have medical insurance. The medical insurance coverage rate is 91.81%. Finally, in terms of social integration, the mean value of native perception of the total sample was 2.98. Specifically, for the statement “I feel I am already a native,” 3.12% of the migrants strongly disagreed, 20.83% disagreed, 50.97% agreed and 25.09% strongly agreed.

### Benchmark Regression

The benchmark regression results in columns (1)–(6) of Table 3, show the impact of health education on medical service utilization of migrants for different diseases, and the result in column (7) shows the overall impact. All estimates controlled county-fixed effects. The results in columns (1)–(6) show that health education has a positive in?uence on the medical service utilization of migrants, after controlling for other variables that may affect medical service utilization. The coefficients for health education are statistically significant at the 1% level for all diseases, except jaundice and conjunctival swelling. After receiving health education, the probability of medical service utilization by the migrants increased by 4.5, 3.5, 5.5, and 5.5% after having diarrhea, fever, rash, and cold, respectively. As shown in column (7), from the general view of medical service utilization, the coefficient of health education is also statistically significant at the 1% level. Receiving health education led to a 5.2% average increase in the medical service utilization of the migrants after an illness, with other factors stay unchanged. The above results indicate that health education promotes the medical service utilization of migrants.

**Table 3:** The effect of health education on medical service utilization of the migrants.

**Variable**	**(1)**	**(2)**	**(3)**	**(4)**	**(5)**	**(6)**	**(7)**
	**Diarrhea**	**Fever**	**Rash**	**Jaundice**	**Conjunctival swelling**	**Cold**	**Overall**
Health education	0.045^***^	0.035^***^	0.055^***^	0.075	0.040	0.055^***^	0.052^***^
	(0.008)	(0.010)	(0.020)	(0.150)	(0.026)	(0.004)	(0.004)
Gender	−0.034^***^	−0.025^***^	−0.059^***^	−0.050	−0.066^***^	−0.015^***^	−0.023^***^
	(0.007)	(0.008)	(0.016)	(0.083)	(0.022)	(0.004)	(0.004)
Age	0.000	−0.004^***^	−0.004^***^	0.008^*^	−0.001	−0.002^***^	−0.002^***^
	(0.000)	(0.001)	(0.001)	(0.004)	(0.001)	(0.000)	(0.000)
Education	−0.003^**^	0.002	0.003	0.013	0.002	−0.001	−0.000
	(0.001)	(0.002)	(0.003)	(0.011)	(0.004)	(0.001)	(0.001)
CPC identity	0.013	0.002	−0.010	0.964^***^	−0.133^***^	−0.001	0.005
	(0.016)	(0.019)	(0.035)	(0.179)	(0.045)	(0.008)	(0.008)
Secondary industry	−0.010	0.015	−0.046	−0.069	0.001	−0.024^*^	−0.033^**^
	(0.026)	(0.029)	(0.055)	(0.139)	(0.066)	(0.014)	(0.014)
Tertiary industry	−0.032	−0.010	−0.063	−0.088	−0.004	−0.051^***^	−0.058^***^
	(0.026)	(0.029)	(0.054)	(0.144)	(0.065)	(0.013)	(0.013)
Marital status	−0.003	0.014	0.028	−0.163	−0.021	0.009	0.005
	(0.011)	(0.013)	(0.026)	(0.125)	(0.034)	(0.006)	(0.006)
Family size	0.000	0.006	−0.003	0.010	−0.013	0.001	0.001
	(0.004)	(0.004)	(0.008)	(0.036)	(0.011)	(0.002)	(0.002)
Hukou	0.020^**^	0.010	−0.016	0.128	0.067^**^	0.024^***^	0.019^***^
	(0.010)	(0.012)	(0.022)	(0.085)	(0.030)	(0.005)	(0.005)
Household income	−0.000	0.001	0.001	−0.008	0.003	0.001^*^	0.001^***^
	(0.001)	(0.001)	(0.001)	(0.011)	(0.002)	(0.000)	(0.000)
Medical insurance	0.039^***^	0.053^***^	0.102^***^	0.215	0.048	0.022^***^	0.027^***^
	(0.013)	(0.016)	(0.031)	(0.161)	(0.040)	(0.007)	(0.007)
Native perception	0.017^***^	0.009	0.020^*^	0.032	0.039^***^	0.014^***^	0.010^***^
	(0.004)	(0.005)	(0.009)	(0.043)	(0.012)	(0.002)	(0.002)
County fixed effects	Yes	Yes	Yes	Yes	Yes	Yes	Yes
Constant	0.243^***^	0.506^***^	0.553^***^	0.122	0.326^***^	0.353^***^	0.428^***^
	(0.040)	(0.046)	(0.088)	(0.322)	(0.113)	(0.020)	(0.020)
Observations	17,560	14,981	4,699	121	2,782	72,911	77,837
R-squared	0.116	0.156	0.201	0.716	0.260	0.098	0.088

*The numbers in parentheses are robust standard errors*.

***, **, and * *represent significance at the 1, 5, and 10% levels, respectively. The following are the same*.

About the results of the control variables in column (7) of Table 3, the effects of the control variables are mostly in line with our expectations, and are consistent with previous studies. Compared to women, men use fewer medical services when they get sick. The effect of age on the medical service utilization of migrants is significant, but small. Compared to the primary industry, working in the secondary and tertiary industries reduces the utilization of medical services by migrants. Education, CPC identity, marital status, and family size affect medical services in the same direction as expected and did not significantly affect the medical service utilization of migrants. Rural Hukou depresses the utilization of medical services by migrants. Similarly, to a certain extent, household income improves the utilization of medical services by migrants. We find that the coefficient of medical insurance is positive and statistically significant at the 1% level, which means that migrants with medical insurance use medical services more. In addition, migrants with higher levels of social integration use more medical services.

### Solving the Problem of Endogeneity

Although the model in this study controls the observable factors that affect the utilization of medical services for migrants, there may still be some unobservable variables that will affect the utilization of medical services for migrants and are related to whether the migrants receive health education. These missing variables lead to biased estimates in this study. This study uses an instrumental variable (IV) model to solve this endogenous problems and strengthen the reliability of the results. Based on the literature (50), this study applies the average number of fixed health education bulletin boards in communities within a county as the IV. Based on the question “Number of fixed health education bulletin boards in the community” in the CMDS community questionnaire, we set the variable of the average number of fixed health education bulletin boards. Theoretically, the average number of fixed health education bulletin boards in communities within a county is a qualified IV that satisfies the requirements of relevance and exclusion. The average number of fixed health education bulletin boards in communities within a county reflects how the communities in the county, where the migrants live, value health education and the popularization of health knowledge, which affects the health education they receive. This indicates that the IV we choose is closely correlated with the explanatory variable. Further, the average number of fixed health education bulletin boards in communities within a county, does not directly associate with the medical service utilization of migrants. Therefore, it qualifies the requirement of exclusion. However, since the 2017 CMDS community data were not open access, we used the 2018 data instead, and the tests illustrate that the IV is still qualified.

The result of the IV are presented in Table 4. After regression with IV, we performed a series of tests. The unidentifiability test showed that the Kleibergen-Paap rk LM was 198.294, rejecting the original hypothesis of unidentifiability. The weak identification test showed that the Kleibergen-Paap rk Wald *F*-value was 201.484, much greater than the empirical criterion 10 and greater than all thresholds, confirming that the average number of fixed health education bulletin boards in communities within a county is a strong IV for health education. These results indicate that the proposed IV is reliable. The result of IV regression is also statistically significant at the 1% level, leading to the conclusion that health education for migrants effectively promotes their utilization of medical services after an illness, a finding consistent with the basic regression.

**Table 4:** The effect of health education on medical service utilization of the migrants (IV model).

**Variable**	**Medical service utilization**
Health education	0.580^***^
	(0.093)
Control variables	Controlled
County fixed effects	No
Observations	69,079

*The control variables are consistent with model 1 and because of space limitations, there is no report here*.

***, ^**^, and ^*^ represent significance at the 1, 5, and 10% levels, respectively.

### Robustness Test

To further enhance our confidence in the research findings, we perform robustness tests by using the logit model and the alternating dependent variable.

#### Logit Model

As the dependent variable of this study, medical service utilization is binary. We further examine the effect of health education on medical service utilization of the mobile population using a logit model. Table 5 shows the result of the logit regression presented as odds ratio. It can be found that controlling for other conditions, the probability of using medical services after an illness significantly increased for the migrants who received health education than those who did not. This is consistent with the findings of the benchmark regression.

**Table 5:** The effect of health education on medical service utilization of the migrants (Logit model).

**Variable**	**Medical service utilization**
Health education	1.271^***^
	(0.025)
Control variables	Controlled
County fixed effects	Yes
Observations	77504
Log-likelihood	−48747.418
Chi2	5629.338

*The coefficient is presented as odds ratio. The control variables are consistent with model 1 and due to space limitations, there is no report here*.

***, ^**^, and ^*^ represent significance at the 1, 5, and 10% levels, respectively.

#### Alternate Dependent Variable

Further, to increase the robustness of the findings, we replaced the dependent variable in this study. Based on the question “Where did you go first for medical services the last time you were sick, injured, or unwell?” in the questionnaire, we constructed a new dependent variable to measure the medical service utilization of migrants. If the respondent was treated at a local community health service station, individual clinic, general/specialized hospital, pharmacy, hometown or another place, the respondent was coded as “1,” defining that medical service utilization occurred. If the respondent answered “No treatment” the respondent was coded as “0,” representing that no medical service utilization occurred. The benchmark, IV, and logit regression results after replacing the dependent variable are shown in columns (1)–(3) of Table 6, respectively.

**Table 6:** The effect of health education on medical service utilization of the migrants (Alternate dependent variable).

**Variable**	**(1)**	**(2)**	**(3)**
	**Benchmark**	**IV**	**Logit model**
Health education	0.025^***^	0.702^***^	1.193^***^
	(0.004)	(0.085)	(0.032)
Control variables	Controlled	Controlled	Controlled
County fixed effects	Yes	No	Yes
Constant	0.773^***^	0.435^***^	0.690
	(0.017)	(0.046)	(0.473)
Observations	62,197	56,357	58,564
R-squared	0.089	–	–
Log-likelihood	–	–	−25814.399
Chi2	–	–	3731.745

*The control variables are consistent with model 1 and because of space limitations, there is no report here. The coefficient of logit model in column and is presented as odds ratio*.

***, ^**^, and ^*^ represent significance at the 1, 5, and 10% levels, respectively.

It can be observed from column (1) of Table 6 that after controlling for other variables of the migrants and county-fixed effects, the coefficient of health education is positive and still statistically significant at the 1% level. We also estimated with an IV, which is the average number of fixed health education bulletin boards in communities within a county. As column (2) of Table 6 shows, the coefficient of health education remained positive and statistically significant at the 1% level. Finally, logit regression analysis was performed. The odds ratio for health education in column (3) of Table 6 is similar to Table 5, and remains statistically significant at the 1% level. Overall, according to the results of the robustness tests reported in Tables 5, 6, health education for migrants enhances their utilization of medical services after an illness. Therefore, the regression results reported in Table 3 are robust.

### Further Exploration on the Methods of Health Education

We attempted to explore the different impacts of educational methods on the medical service utilization of migrants. The migrants in China received health education through health lectures, promotion materials (paper or video), bulletin boards and electronic displays, community SMS/WeChat/website, public health consultation, and individual consultation. We classified public health and individual counseling as counseling, and other methods of health education as non-counseling. Table 7 presents the results. The coefficients for both types of health education methods were positive and significant at the 1% level, indicating that either method of health education can improve the medical service utilization of the migrants. However, the promotion effect of counseling is more greater. In summary, compared to other health education methods, counseling is more effective in improving the medical service utilization of migrants.

**Table 7:** The effect of different health education methods on medical service utilization of the migrants.

**Variable**	**(1)**	**(2)**
	**Non-counseling**	**Counseling**
Health education	0.028^***^	0.079^***^
	(0.005)	(0.005)
Control variables	Controlled	Controlled
County fixed effects	Yes	Yes
Constant	0.436^***^	0.426^***^
	(0.025)	(0.025)
Observations	48,438	48,880
R-squared	0.094	0.104

*The control variables are consistent with model 1 and because of space limitations, there is no report here*.

***, ^**^, and ^*^ represent significance at the 1, 5, and 10% levels, respectively.

### Moderating Effect Analysis

Based on the results of the benchmark regression and the robustness test, we are confident that health education has a positive effect on the medical service utilization of migrants. To explore whether other factors influence the effect of health education on medical service utilization, we conducted a moderating effect analysis.

Based on the literature, many barriers prevent migrants from medical services (51, 52), and distance and transportation are important factors (53, 54). Thus, we hypothesized that the effect of health education on the medical service utilization of migrants is moderated by the accessibility of medical services. Based on the question “How long does it take to go from your residence to the nearest medical service institution?” in the questionnaire, we constructed a variable for the accessibility of medical services as a moderator variable. There are four options for this question: “within 15 min,” “15–30 min,” “30–60 min,” and “more than 1 h.” We coded “within 15 min” as “1,” defining the accessibility of medical services, and the other options as “0.” We added the interaction term of the explanatory variable (health education), and moderator variable (the accessibility of medical services), to the regression model. The result of the moderating effect analysis are presented in Table 8. It can be found that controlling for other variables and county fixed effects, the coefficient of the interaction term of health education and the accessibility of medical services is positive and statistically significant at the 1% level. This demonstrates that the accessibility of medical services positively moderates the effect of health education on medical service utilization for migrants. That is to say, health education can improve the utilization of medical services of migrants, and the closer the migrants are to medical service institutions, the stronger the effect of health education on migrants' medical services utilization.

**Table 8:** Moderating effect of accessibility in the effect of health education on medical service utilization of the migrants.

**Variable**	**Medical service utilization**
Health education	0.036^***^
	(0.007)
Health education*Accessibility	0.019^***^
	(0.006)
Control variables	Controlled
County fixed effects	Yes
Observations	77,837
R-squared	0.088

*The control variables are consistent with model 1 and because of space limitations, there is no report here*.

***, ^**^, and ^*^ represent significance at the 1, 5, and 10% levels, respectively.

### Heterogeneous Impact Analysis

We further explore the heterogeneous impact of health education on the medical service utilization of migrants in different groups. We divided the total sample into subsamples according to age and education level, and used the basic analysis model. The results are listed in Table 9.

**Table 9:** The effect of health education on medical service utilization of the migrants on different groups.

**Variable**	**(1)**		**(2)**	
	**Age**		**Education**	
	**Non-elderly**	**Elderly**	**Non-higher**	**Higher**
Health education	0.053^***^	0.044	0.055^***^	0.039^***^
	(0.004)	(0.041)	(0.005)	(0.010)
Control variables	Controlled	Controlled	Controlled	Controlled
County fixed effects	Yes	Yes	Yes	Yes
Constant	0.339^***^	0.392^***^	0.404^***^	0.536^***^
	(0.018)	(0.144)	(0.020)	(0.052)
Observations	76,621	935	62,472	15,205
R-squared	0.086	0.423	0.099	0.112
Empirical *P*-value	0.010^**^	0.002^***^		

*The control variables are consistent with model 1 and because of space limitations, there is no report here. The “Empirical P-value” is used to test the significance of the difference in the coefficients of health education between groups, obtained by self-sampling 500 times through bootstrapping*.

***, **, and ^*^ represent significance at the 1, 5, and 10% levels, respectively.

Referring to studies on aging, we classify the migrants who are over 60 years old as elderly and code them as “1,” and the migrants who are <60 years old are coded as “0” (55, 56). The result in column (1) of Table 9 indicate that the effect of health education on the medical service utilization of migrants is heterogeneous in different age groups. The effect of health education on medical service utilization among elderly migrants is not significant, and the effect is greater and significant at the 1% level for non-elderly migrants. The heterogeneous effect between age groups was significant at the 5% level through Fisher's permutation test. This indicates that health education has a stronger impact on the medical service utilization of non-elderly migrants.

Further, we explored the impact of health education on migrants with different levels of education. We coded “undergraduate/junior college” and above as “1,” indicating higher education (57), and others as “0.” The result in column (2) of Table 9 similarly indicate that there is a heterogeneous effect in education level groups. Specifically, the effect of health education on medical service utilization for both migrants with and without higher education is significant at the 1% level, but greater for migrants who did not receive higher education. Fisher's permutation test also demonstrates that the differences here are not coincidental, and health education is more effective in promoting medical service utilization of migrants without higher education. Compared with migrants who have received higher education, those with a low level of education have relatively poorer health literacy, and thus, health education will have a greater effect.

## Discussion

### Main Findings

Improving medical service utilization among the migrants can improve the health of the migrants and reduce health inequalities. The low utilization of health services among the migrants compared to residents is not only caused by the fragmentation of the social insurance system, but also related to the migrants' lack of health literacy. The Chinese government has taken steps to provide health education to migrants. This study examined whether health education effectively improves the health literacy of migrants, and thereby their medical service utilization.

In this study, based on a large sample of data from CMDS, we used multiple linear regression, logit regression, and instrumental variable methods to examine the effect of health education on medical service utilization among migrants and the different effects of different health education methods. We further tested the moderating effect of the accessibility of medical services and the heterogeneous effects of health education on the medical service utilization of migrants belonging to different age and education groups. We provide empirical evidence for further improving medical service utilization and conducting more effective health education among migrants.

We find that migrants significantly use more medical services after receiving health education, and this result is significant while controlling migrants' household income and medical insurance. In general, low income and the fragmentation of the social insurance system are the main reasons for the low utilization of medical services by migrants (58, 59). However, what is not considered is the low health literacy of the migratory population. Most migrants come from under-developed rural areas, have a heavy burden of life, poor hygiene, low health literacy, and limited knowledge about diseases. Thus, health problems do not attract their attention, and they often do not take any measures when they fall ill. After receiving health education, firstly, migrants gain knowledge about diseases and health, learn about the harm of diseases, and value their health more. Secondly, by receiving health education locally, migrants can obtain information about local access to medical services, which makes them more familiar with how to use medical services in the inflow city easily and quickly and reduces the cost of accessing medical services. As a result, the medical services utilization of migrants will be improved.

Our findings further elucidate how to conduct health education more efficiently. In practice, migrants receive many forms of health education, divided into counseling and non-counseling types. Compared to non-counseling, counseling health education is more effective in promoting medical service utilization among migrants. Through public health and personal counseling, migrants have access to one-to-one health advice, which is more efficient in health knowledge transfer. In addition, migrants can get consultations regarding their health problems and hygiene, to obtain the most compatible medical diagnosis and health care advice, and better matched health knowledge, which is more effective in improving their health literacy.

We also find that the accessibility of health care services moderates the effect of health education on medical service utilization among migrants. The closer the migrants are to medical service institutions, the stronger the effect of health education on their medical service utilization. After receiving health education, migrants make more use of medical services when they are ill. However, if migrants do not have easy access to medical services—for example, if they are too far away from a healthcare facility—it makes it more difficult for them to access medical services and further discourage them from seeking medical services, which will eventually weaken the effect of health education on migrants' medical service utilization. Conversely, if they have easy access to medical services, they will be more motivated to use medical services when they are sick.

From the age perspective, health education has a stronger effect on promoting medical service utilization among non-elderly migrants than elderly migrants. Possible reasons for this are that elderly migrants are more concerned about health issues as their physical health declines with age, and the same diseases cause more financial and health losses. On the other hand, in China, non-elderly migrants are the mainstay of their family, and bear more familial and economic responsibilities, which forces them to focus more on increasing their income rather than their health. Therefore, health education is more effective in increasing the non-elderly mobile population's attention to their health and improving their medical service utilization.

From the perspective of education, health education has a stronger effect on promoting medical service utilization among migrants with lower education levels. This is because, compared to migrants who are not well educated, migrants with undergraduate/junior college degrees and above, generally have higher health literacy (60) and pay more attention to their health problems. Hence, the effect of health education on them is limited. However, most migrants are not highly educated, have relatively poor health knowledge, and lack health awareness. Therefore, health education can better improve health literacy for migrants who not well educated, and increase their attention to health issues and medical service utilization.

### Policy Implications

Based on the findings of our study, we offer some suggestions for reference. Migrants, communities, and the government should be aware of the importance of health education in improving medical service utilization. For migrants, they should primarily pay attention to their health problems, for their good health is their important human capital (61), and it affects their social capital (62, 63). Second, migrants should recognize that their health literacy is relatively low, so they should participate more actively in health education to accumulate more health-related knowledge, improve their health literacy, and actively use medical services for treatment when they are ill. Communities should fulfill their responsibilities of primary-level governance and focus on the health of migrants. They should work together with medical service providers to organize health education for migrants, especially public health counseling and individual counseling, which are more effective in improving the utilization of medical services.

For the government, to improve medical service utilization and reduce health inequalities among migrants, efforts should be made to develop supporting policies to promote the health of migrants, improve the accessibility of medical services, strengthen the propaganda of relevant policies and health education, and conduct health education precisely. First, promote the formation of a policy environment conducive to the medical service utilization of migrants. Health department and other departments should coordinate to form policies that facilitate the migrants' access to medical services, include them in the scope of community health services, and consider them when formulating and revising policies on health education, disease prevention, treatment, and medicine. Second, improve the accessibility of medical services for migrants. The government needs to optimize the allocation of medical service resources, innovate the service model, improve the accessibility of medical services, and ensure that the migrants have convenient access to medical services, in response to the characteristics of the local migrants' work and residence, the major health problems and health needs they face. Third, strengthen the promotion of health education and medical service policies. The government can use appropriate media forms and seek help from labor unions, associations, and non-governmental organizations, to make migrants more familiar with information regarding health education and medical service policies, as well as the contents and processes of medical services. Finally, health education could be more precise and effective. The government should keep improving the health education service model, actively carry out health education for migrants of different ages, genders, and occupations, and especially pay attention to the non-elderly and less educated migrants. It should also develop more consulting-type health education, to continuously improve efficiency regarding the enhancement of the health literacy of migrants.

### Strengths and Limitations

In this study, we evaluate the effect of health education on the medical service utilization of migrants, and offer three main contributions. First, this study uses a large sample of data to empirically examine the effect of health education on improving medical service utilization among migrants. This is an important addition to the current literature on enhancing medical service utilization among migrants. Second, we studied the effect of different health education methods on improving medical service utilization among migrants, which has important practical implications for governments on how to provide better health education, and reduce health inequities. Third, this study also explores the moderating effect of the accessibility of medical services, and the heterogeneous effects of health education on medical service utilization by migrants belonging to different age and education groups; this helps in gaining a comprehensive understanding of the role of health education in enhancing medical service utilization among migrants. It also allows governments to develop better policies to alleviate health inequalities among mobile populations.

However, this study has some limitations. First, this study only investigates the effect of health education on the medical service utilization of migrants. It does not explore other important factors, such as the fragmentation of the insurance system and how to integrate. Second, due to the limitation of data, this study uses 2017 cross-sectional data. Subsequent studies with panel data would make the causal inference more robust and reliable.

## Conclusion

Migrants have made significant contributions to China's economic and social development, yet suffer from health inequities, particularly the low utilization of medical services. This study shows that health education could improve the medical service utilization of migrants, and the effect is stronger if a counseling-type health education is conducted, or if migrants have better access to health services. The increase in medical service utilization was more prominent among non-elderly and low-education migrants after health education. In future, we will continue to explore the causes affecting medical service utilization among migrants from different demographics, to provide support for reducing health inequalities. In addition, tracking panel data could better answer the above questions and make the conclusions of causal inference more reliable.

## Data Availability Statement

Publicly available datasets were analyzed in this study. This data can be found here: China Migrants Dynamic Survey https://chinaldrk.org.cn/wjw/#/home.

## Author Contributions

YT led and designed the study, led the data collection, analysis, and interpretation. TL contributed to the study design, provided input into the data analysis, and wrote the first draft of the manuscript. YC contributed to the study design, data interpretation, reviewed the manuscript, and helped the writing of the final draft manuscript. All the authors read and approved the final manuscript.

## Funding

This paper was funded by Full-time postdoctoral research and development fund project of Sichuan University, Research on the Accurate Configuration of Medical Public Services Empowered by Smart Technology in the Post-epidemic Era (skbsh2020-05), Independent project of School of Public Administration of Sichuan University, Research on the Accurate Supply of Medical Public Services Empowered by Big Data in the Post-epidemic Era (2020Ziyan-gongguan05), the 2020 Project of the Social Development and Social Risk Control Research Center of Sichuan Philosophy and Social Sciences Key Research Base, Research on Accurate Community Emergency Management Under the Background of Big Data (SR20A09) and the 2021 Project of the Central Universities Basic Research Funds for School of Public Administration of Sichuan University, the Development History and Future Prospects of Ethnic Higher Education under the Leadership of the Communist Party of China (2021DSDJ007).

## Conflict of Interest

The authors declare that the research was conducted in the absence of any commercial or financial relationships that could be construed as a potential conflict of interest.

## Publisher's Note

All claims expressed in this article are solely those of the authors and do not necessarily represent those of their affiliated organizations, or those of the publisher, the editors and the reviewers. Any product that may be evaluated in this article, or claim that may be made by its manufacturer, is not guaranteed or endorsed by the publisher.

## Abbreviations

IV: instrumental variable
CMDS: china migrants dynamic survey.
